# Deep convolutional neural networks for automatic segmentation of thoracic organs‐at‐risk in radiation oncology – use of non‐domain transfer learning

**DOI:** 10.1002/acm2.12871

**Published:** 2020-06-29

**Authors:** Charles C. Vu, Zaid A. Siddiqui, Leonid Zamdborg, Andrew B. Thompson, Thomas J. Quinn, Edward Castillo, Thomas M. Guerrero

**Affiliations:** ^1^ Beaumont Artificial Intelligence Research Laboratory Beaumont Health System, Royal Oak MI USA; ^2^ Department of Radiation Oncology Beaumont Health System, Royal Oak MI USA

**Keywords:** convolutional neural network, organs‐at‐risk, segmentation, transfer learning

## Abstract

**Purpose:**

Segmentation of organs‐at‐risk (OARs) is an essential component of the radiation oncology workflow. Commonly segmented thoracic OARs include the heart, esophagus, spinal cord, and lungs. This study evaluated a convolutional neural network (CNN) for automatic segmentation of these OARs.

**Methods:**

The dataset was created retrospectively from consecutive radiotherapy plans containing all five OARs of interest, including 22,411 CT slices from 168 patients. Patients were divided into training, validation, and test datasets according to a 66%/17%/17% split. We trained a modified U‐Net, applying transfer learning from a VGG16 image classification model trained on ImageNet. The Dice coefficient and 95% Hausdorff distance on the test set for each organ was compared to a commercial atlas‐based segmentation model using the Wilcoxon signed‐rank test.

**Results:**

On the test dataset, the median Dice coefficients for the CNN model vs. the multi‐atlas model were 71% vs. 67% for the spinal cord, 96% vs. 94% for the right lung, 96%vs. 94% for the left lung, 91% vs. 85% for the heart, and 63% vs. 37% for the esophagus. The median 95% Hausdorff distances were 9.5  mm vs. 25.3 mm, 5.1  mm vs. 8.1 mm, 4.0  mm vs. 8.0 mm, 9.8  mm vs. 15.8 mm, and 9.2 mm vs. 20.0 mm for the respective organs. The results all favored the CNN model (*P* < 0.05).

**Conclusions:**

A 2D CNN can achieve superior results to commercial atlas‐based software for OAR segmentation utilizing non‐domain transfer learning, which has potential utility for quality assurance and expediting patient care.

## 1 INTRODUCTION

Accurate delineation of tumor volumes and organs‐at‐risk (OARs) is an essential component of the radiation oncology workflow. In treatment planning for thoracic malignancies, such as lung cancer, esophagus cancer, or breast cancer, commonly segmented thoracic OARs include the heart, esophagus, spinal cord, and lungs. Accurate delineation of thoracic OARs is necessary to assess the dose to these critical normal structures.

Numerous methods have been attempted for automatic segmentation of thoracic organs, including atlas‐based methods, level‐set methods, and morphological methods (see Sharp et al.^1^ for a detailed review). Deep convolutional neural networks (CNNs) have revolutionized numerous areas of medical image analysis. For image classification problems, CNNs have achieved human‐level results in classification of skin cancer and diabetic retinopathy.^2^, ^3^ While CNNs initially achieved state‐of‐the‐art results in image classification tasks, their use in semantic segmentation was initially proposed by Long et al.^4^ Subsequently, there have been a series of incremental improvements to the state‐of‐the‐art in semantic segmentation. Subsequently, deep CNNs have also achieved state‐of‐the‐art results in medical image segmentation problems, such as magnetic resonance imaging‐based segmentation of the brain and prostate.^5^, ^6^ The most widely used architecture for segmentation tasks is the U‐net model. In this architecture, an image is processed by successive convolutional layers down to a bottleneck layer, similar to the design of a network for a classification task. Given that the output of the image is required to be a segmentation map at input resolution rather than merely a single classification, the bottleneck layer is then up‐sampled back with deconvolutions until a pixel‐wise classification is achieved at input resolution.

Previous studies attempting thoracic auto‐segmentation using CNNs have used relatively small datasets for model development,^7^, ^8^ but these small datasets are often not amenable to good performance with deep learning models. As such, we attempted to utilize a larger dataset obtained from routine clinical use to efficiently train a 2D model for the task of semantic segmentation. In order to further improve model convergence, we hypothesized that utilizing transfer learning from a model pre‐trained on the ImageNet classification task would be beneficial.^9^ We therefore modified the classic U‐Net model, replacing the downward convolutional path with the VGG16 network,^9^ which has a publically available model with weights pre‐trained on ImageNet. We hypothesized that our methodology would allow for convergence of a 2D neural network model with acceptable accuracy, low GPU overhead, and fast inference times.

## 2 MATERIALS AND METHODS

### 2.A Training dataset

A data preprocessing system was engineered to automatically import treatment plans from a clinically deployed Pinnacle (Philips, Amsterdam) treatment planning system (TPS). This preprocessing pipeline converted the TPS contour format to industry‐standard DICOM RTSTRUCT format. A region‐of‐interest vocabulary (right lung, left lung, heart, spinal cord, and esophagus) and synonym lists (e.g., R_lung, lung_R, etc as valid names for right lung) were developed in coordination with dosimetry staff to ensure consistency between patient plans. No registration was performed on input images. All contours were used as part of clinical treatment planning and were assumed to represent the ground truth. The contours were not adjusted or changed for this study. The resulting dataset consisted of axial CT slices and corresponding pixel label maps.

The training set is comprised of the volumetric CT images and corresponding contour information used for radiation therapy simulation and treatment planning for 168 patients. The data were extracted as part of an Institutional Review Board‐approved retrospective study (patient consent waived due to minimal risk). The dataset was split into training, validation, and test sets, with all slices from a patient’s CT scan belonging exclusively to one of the three groups.

OAR contours were rasterized as label images using region‐filling techniques. Pixel‐wise classification of images was used, such that no overlapping ROIs were permitted. CT slice data were down‐sampled to half‐resolution (256 x 256) due to GPU capacity limitations. As the model then produced pixel‐wise classifications at half‐resolution (256 x 256 pixels), we up‐sampled the output from the GPU to full resolution (512 x 512 pixels) using nearest‐neighbor interpolation for statistical analysis. The training data was normalized to mean value zero and the validation and test datasets were subtracted by the mean pixel value of the training dataset prior to inference.

### 2.B Convolutional neural network structure

**Fig. 1:**
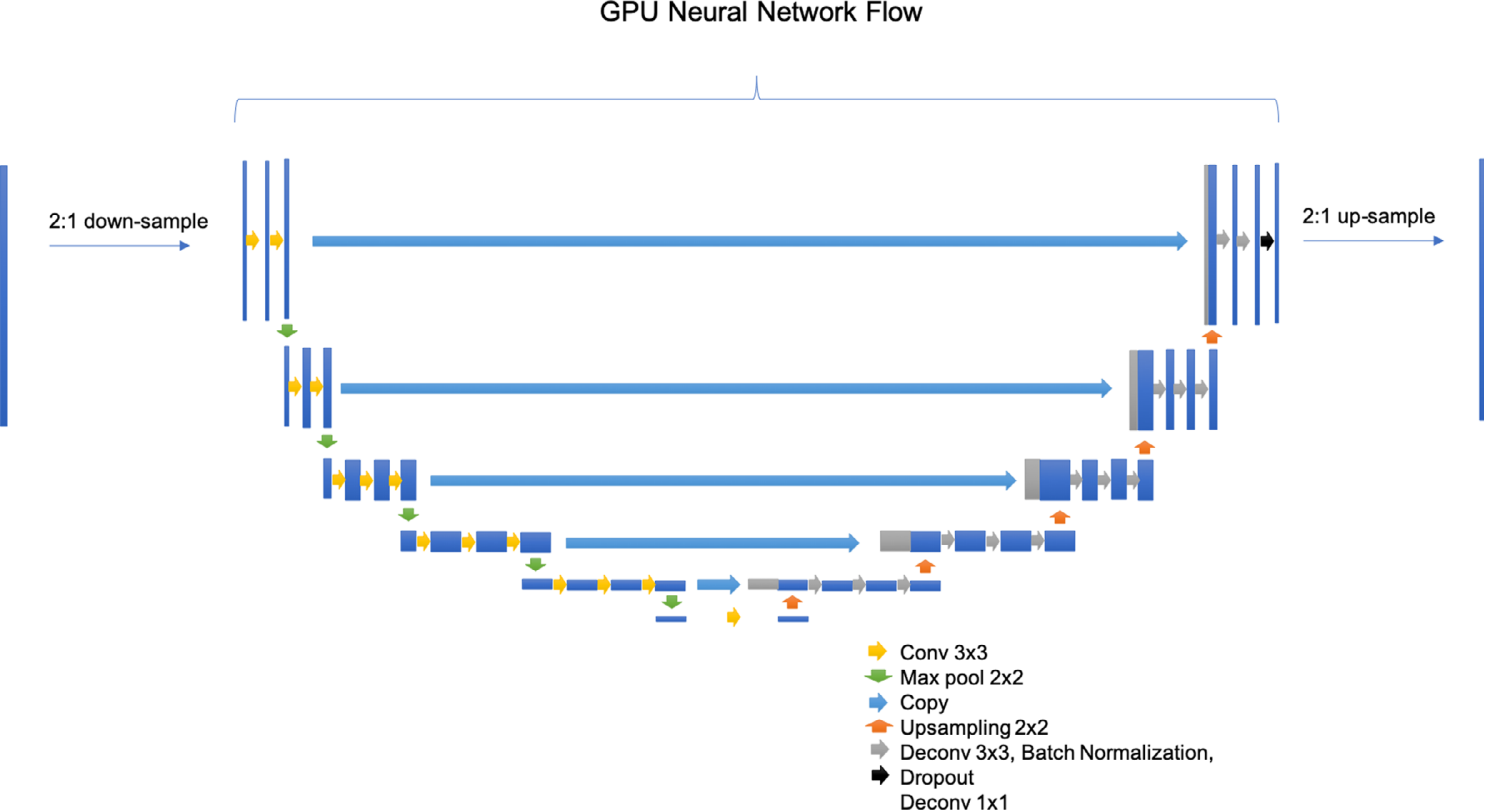
Convolutional Neural Network Structure (modified U‐Net, adapted from Ronneberger et al. [15]). The input CT slice is down‐sampled due to GPU memory limitations. The downward path is the VGG16 model from keras trained on ImageNet with locked weights. The upward path mirrors the VGG16 path with some modifications to enable faster convergence. Activation functions not shown for clarity. The output of then neural network is then up‐sampled for the full resolution prediction. Up‐sampling and down‐sampling is done with 2:1 nearest neighbor resizing (both for pre/post‐processing and within the GPU neural network flow).

We used a modified U‐Net, adapted from the original U‐Net structure from Ronneberger et al.^10^ Implementation was done using Tensorflow and Keras. The model architecture is shown in Fig. 1. For the encoder section of the U‐net (the downward path), the unmodified VGG16 network was used from Keras. We applied transfer learning by using locking the weights from a VGG16 image classification model trained on ImageNet^9^ available from the Keras API. Utilizing the same model without locked pre‐learned weights resulted in failure to converge (data not shown). For the upward path, we mirrored the downward path with up‐sampling kernels and deconvolution kernels, but unlike the downward path we modified the activation function to be a leaky rectified linear units (leaky ReLU) activation. In addition, after every leaky ReLU activation layer, we utilized a batch normalization layer^11^ and a dropout layer,^12^ with a dropout coefficient of 0.25. These changes were made to regularize the network and allow faster convergence without overfitting.

The model was trained end‐to‐end online on four NVIDIA K80 GPUs, utilizing the ADAM optimizer with an initial learning rate of 0.5 to minimize the categorical cross‐entropy loss function.^13^ Data augmentation was not utilized in our model. No post‐processing or ensemble training was utilized.

### 2.C Evaluation metrics

To assess the accuracy of contours, the Dice similarity coefficient and average Hausdorff distance were calculated for each contour in the test dataset.^14^, ^15^ The SlicerRT extension of the 3D Slicer program (http://www.slicer.org) was utilized to calculate evaluation metrics on the test dataset.^16^ Box plots were generated using R Studio (version 1.2.1335) to measure the patient‐level variability in Dice coefficients between subjects in the test dataset.

We also compared our contours to commercial automatic segmentation software. The atlas segmentation module of MIM version 6.7 (MIM Software, Cleveland, OH, USA) was used to generate thoracic OAR contours on the test dataset. The atlas was comprised of the same patients in the training dataset used to develop our deep learning model. As in our CNN model, no post‐processing was performed on the atlas‐based contours. The Dice similarity coefficient and 95% Hausdorff distances for each model was compared with the two‐tailed Wilcoxon signed‐rank test.

## 3 RESULTS

In total, 22,411 CT slices from 168 patients were included in this study. Patients were divided into training, validation, and test datasets according a 66%/17%/17% split (n = 112/28/28). Model training lasted approximately 2.5 days. Inference time after training was approximately 15 s per patient.

**Fig. 2:**
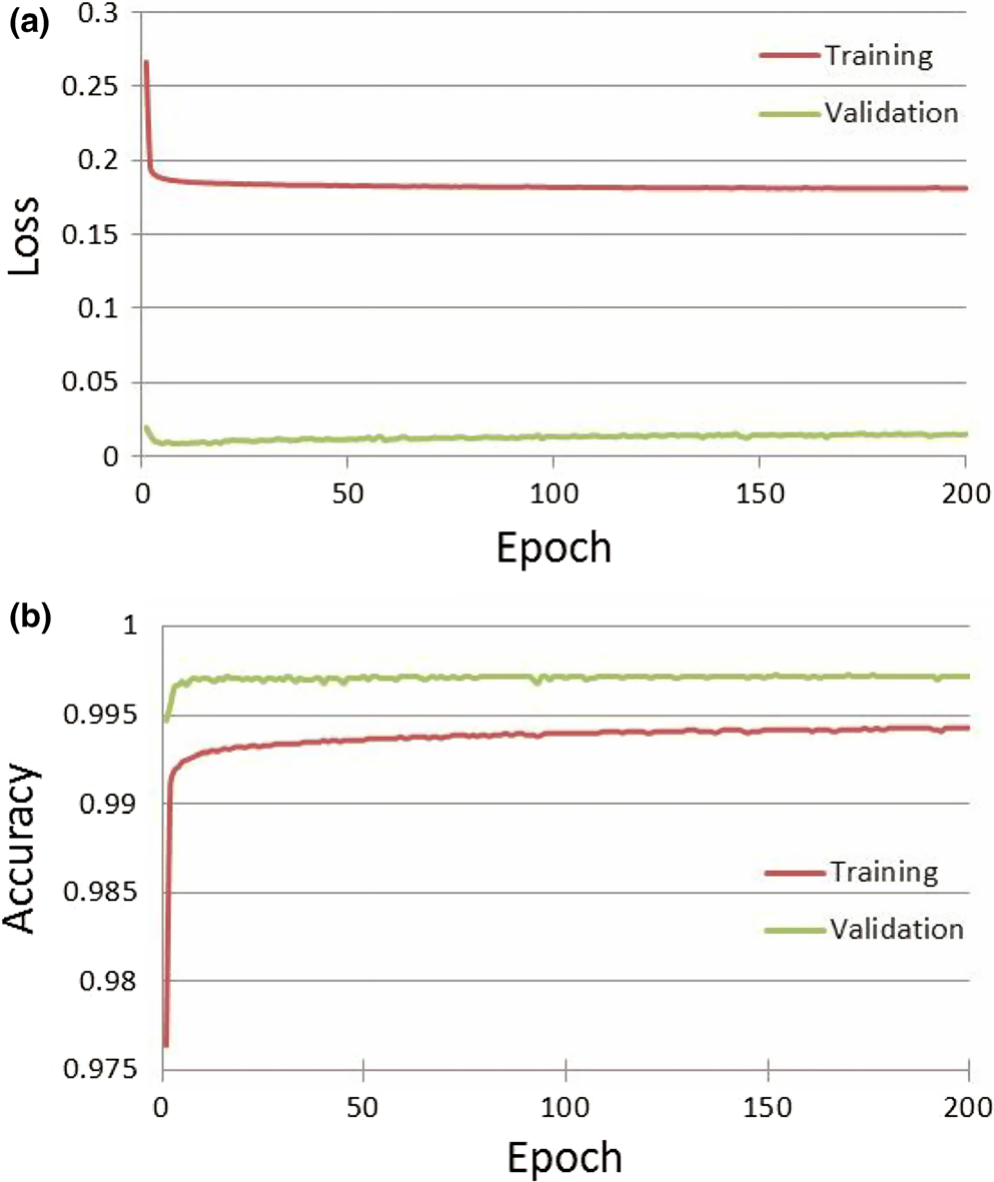
Training and Validation Dataset Loss (a) and Accuracy (b) during Model Training. Panel A shows the loss function (categorical cross‐entropy) decreasing with each epoch of training. Panel B shows the accuracy (Dice) improving with each epoch.

**Fig. 3:**
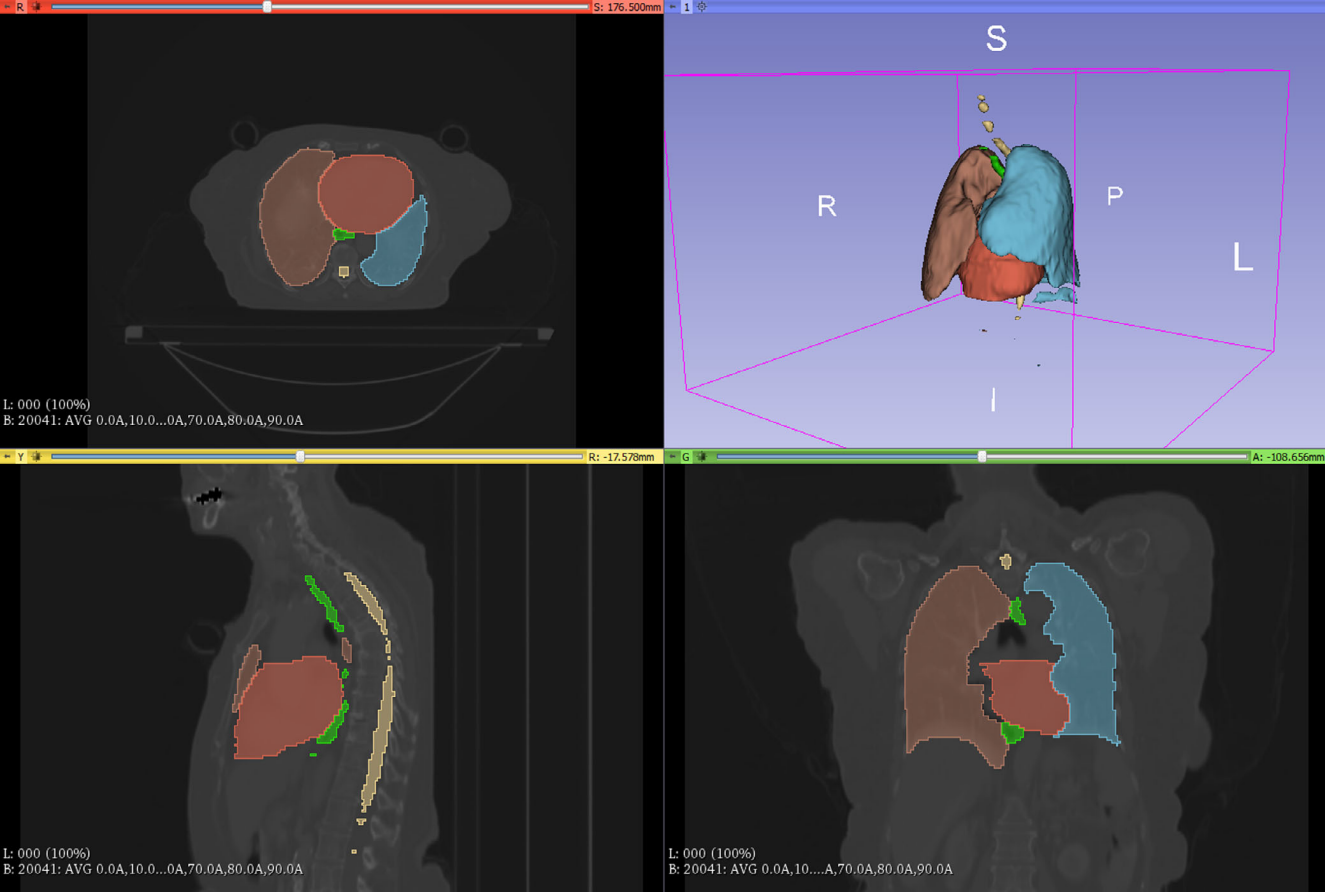
Example Segmentation From Test Dataset Using Convolutional Neural Network Model. Counter‐clockwise from top left: axial view, sagittal view, coronal view, and 3D reconstruction of the five OARs trained (Right lung, left lung, heart, esophagus, and spinal cord).

See Fig. 2 for a plot of the loss and accuracy for the training and validation datasets. The training accuracy is lower than the validation accuracy and the training loss is higher than the validation loss because of the use of dropout and batch normalization as regularization procedures to protect against overfitting during model training. Figure 3 shows a sample segmentation from our model showing all five organs‐at‐risk: right lung, left lung, heart, esophagus, an spinal cord.

On the test dataset, our model (CNN) was compared to the commercial multi‐atlas model algorithm with the same training dataset as input. The mean Dice coefficient of the CNN vs. multi‐atlas model was 75% vs. 63% for the spinal cord (*P* = 0.01), 97% vs. 93% for the right lung (*P* = 0.005), 97% vs. 91% for the left lung (*P* < 0.001), 90% vs. 83% for the heart (*P* < 0.001), and 64% vs. 39% for the esophagus (*P* < 0.001).

The median 95% Hausdorff distances were 9.5 mm vs. 25.3 mm for the spinal cord (*P* = 0.002), 5.1 mm vs. 8.1 mm for the right lung (*P* = 0.002), 4.0 mm vs. 8.0 mm for the left lung (*P* < 0.001), 9.8 mm vs. 15.8 mm for the heart (*P* < 0.001), and 9.2 mm vs. 20.0 mm for the esophagus (*P* < 0.001).

**Fig. 4:**
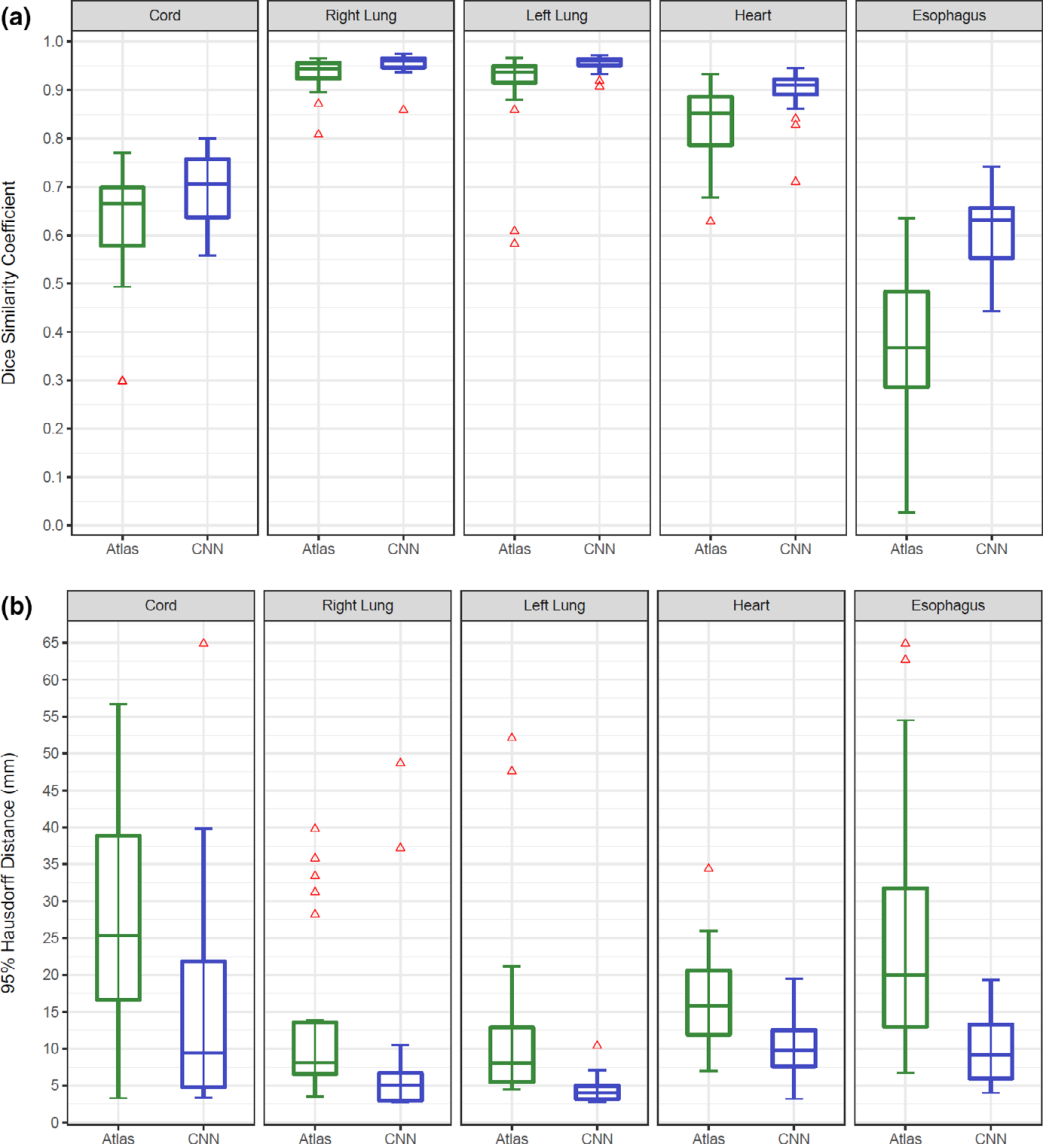
Dice Similarity Coefficient Box Plot (a) and 95% Hausdorff Distances (b) for Atlas‐Based (green) and Convolutional Neural Network (blue) Models. CNN, convolutional neural network. Atlas and convolutional neural network models are compared with the paired Wilcoxon signed rank test showing improved performance with the neural network on all structures.

The distribution of results on the test dataset demonstrates lower variability with the CNN model (See Fig. 4 for boxplot representation).

## 4 DISCUSSION

This paper presents a deep CNN trained on a large dataset of routinely contoured patients. With this model, we were able to achieve superior segmentation results to a commercial atlas‐based algorithm for all five thoracic OARs evaluated in this study. This suggests that utilization of this algorithm would assist in faster contouring, although we did not explicitly test this hypothesis due to concern that differences in user edits could wash out a difference between the underlying algorithms.

Classically, neural networks have required both large computation clusters and large datasets to efficiently train. We were able to get around the first limitation using a 2D model – thereby enabling the use of a consumer‐level GPU. The second limitation was circumvented by utilizing transfer learning from a model that achieved state‐of‐the‐art results on a public image challenge (ImageNet). Despite the original task being unrelated to medical imaging (or even segmentation), this approach allowed our model to reach a high accuracy. A pre‐trained 2D model may mirror human contouring practices more accurately than a 3D model, even if with unlimited GPU capacity, as humans usually contour axially. In addition, most scanners standardize the input dimensions in the x‐y plane (512 x 512 pixels on most modern scanners), but the z‐dimension fields of view and pixel size vary depending on manufacturer and application.

One of the potential benefits of a robust automatic segmentation algorithm is a reduction in contouring time by clinicians. Lustberg et al. evaluated whether atlas and/or deep learning‐based automatic contouring algorithms could reduce the time spent contouring lung cancer OARs[13]. Using 20 patients for training and validation, deep learning contours reduced manual contouring of the spinal cord, lungs, heart, mediastinum, and esophagus from 20 to 10 min. In addition to reducing the time for contouring, utilizing a model for OARs may improve variation in reporting of dose to these organs and thereby improve reporting of dosimety on clinical trials.

As with all studies, the strengths of our work should be weighed against its limitations. First, because the data was obtained from normal clinical operations, the ground‐truth labels were generated by multiple different radiation oncologists leading to intra‐observer variation of the “ground‐truth” labels. Additionally, the borders of the spinal cord were not consistent between patients, likely making it difficult for the model to learn an appropriate representation of this structure. Finally, all patients in this study were treated at a single center on the same CT simulator and it remains to be seen whether such an approach is generalizable to all centers.

In conclusion, we demonstrate that accurate segmentation OARs may be achieved with a 2D U‐ net model with the use of non‐domain transfer learning. Future work will be required to validate the utility of this model in generating OARs robust enough for treatment planning.

## CONFLICTS OF INTEREST

ZAS is a University Ambassador for Nvidia Corp (non‐financial relationship). Nvidia Corp did not participate in any part of this study.
